# Universal access to anti-HIV therapy

**DOI:** 10.1186/1742-4690-9-S1-I13

**Published:** 2012-05-25

**Authors:** Yves Souteyrand

**Affiliations:** 1 HIV/AIDS Department World Health Organization, Geneva, Switzerland

## 

WHO, in collaboration with UNAIDSs and UNICEF, report annually on progress in the different HIV/AIDS interventions. In 2010, more than 6.6 million people had access to Antiretroviral Therapy (ART), in low-and –middle income countries, representing a 17-fold increase in less than 7 years. This is one of the most successful public health achievements.

However, this represents less than 50% coverage of needs. The 2010 global commitment of Universal Access to ART has not been achieved.

The presentation will address determinants of progress and of inequalities in access to ART, according to geographical and population criteria. West and Central Africa as well as Eastern Europe and Central Asia are reporting low coverage rates compared to other similar regions. Coverage among children less than 15 years old is much lower than adult coverage (23% versus 51%). Access to ART for intravenous drug users is very low in most countries. In many settings, access for people in rural areas is much more limited than in public area.

Closing the gap in access to ART will need implementing huge efforts to reduce inequities in access. A comprehensive approach should include improvement of health delivery systems, decentralization of care, simplification of treatment and laboratory monitoring. It will need also to address essential human right issues, including stigmatization and discrimination of key populations who currently do not have access to testing and counseling and other essential interventions.

